# The role of respiratory function tests in infants with stridor: diagnosis at glance and follow-up

**DOI:** 10.1186/s13052-024-01716-8

**Published:** 2024-09-04

**Authors:** Silvia Bloise, Raffaella Nenna, Laura Petrarca, Maria Giulia Conti, Greta Di Mattia, Luigi Matera, Enrica Mancino, Domenico Paolo La Regina, Riccardo Lubrano, Enea Bonci, Corrado Moretti, Fabio Midulla

**Affiliations:** Dipartimento Materno Infantile e di Scienze Urologiche, Sapienza Università di Roma, UOC di Pediatria e Neonatologia Ospedale Santa Maria Goretti, Polo Pontino, Roma, Italy

**Keywords:** Respiratory function testing, Stridor, Infant, Airway obstruction

## Abstract

**Background:**

Recently, the development of advanced, noninvasive methods has allowed the study of respiratory function even in uncooperative infants. To date, there is still little data on the application of this technique in infants with suspected airway obstruction. The aims of our study were:

- To evaluate the role of respiratory function testing (PFR) in the diagnosis and follow-up of infants with stridor

- To evaluate the differences between patients with inspiratory stridor and expiratory stridor.

- To evaluate the concordance between PFR and endoscopy.

**Methods:**

We enrolled infants aged < 1 year with a diagnosis of inspiratory and/or expiratory chronic stridor and a group of healthy controls. For each patient we performed PFR at diagnosis (T0) and for cases at follow-up, at 3 months (T1), 6 months (T2), 12 months (T3). At T0, all patients were classified according to a clinical score, and at follow-up, stature-ponderal growth was assessed.

When clinically indicated, patients underwent bronchoscopy.

**Results:**

We enrolled 48 cases (42 diagnosed with inspiratory stridor and 6 expiratory stridor) and 26 healthy controls. At T0, patients with stridor had increased inspiratory time (*p* < 0.0001) and expiratory time (*p* < 0.001) than healthy controls and abnormal curve morphology depending on the type of stridor. At T0, patients with expiratory stridor had a reduced Peak expiratory flow (*p* < 0.023) and a longer expiratory time (*p* < 0.004) than patients with inspiratory stridor.

We showed an excellent concordance between PFR and endoscopic examination (k = 0.885, *p* < 0.0001). At follow-up, we showed a progressive increase of the respiratory parameters in line with the growth.

**Conclusions:**

PFR could help improve the management of these patients through rapid and noninvasive diagnosis, careful monitoring, and early detection of those most at risk.

## Background

Stridor is a high-frequency musical sound generated by the vibration of obstructed airways as a result of turbulent airflow. Depending on the site of the obstruction, the stridor may be inspiratory, if extrathoracic airway (nose, pharynx, larynx, and trachea) are involved, or expiratory, if the obstruction is located in the intrathoracic airway (tracheobronchial tree) [1, 2].

Stridor can be congenital or acquired. The acquired stridor is generally acute, mainly manifest at after 6 months of age and can be caused by infection, foreign body aspiration and iatrogenic insults; while congenital stridor has a chronic course and tends to occur at birth or in the first few months of life [3]. Laryngomalacia represents the most frequent congenital cause of stridor in the first year of life, 90% of patients are managed conservatively with resolution of symptoms by 12–24 months of age [4–6].

Other causes of stridor can be vocal cord paresis, laryngeal webs, congenital subglottic stenosis, tracheomalacia, tracheal stenosis and subglottic hemangioma [7–11].

To date, the gold standard for the diagnosis in patients with suspected airway obstruction is the airway endoscopic, that is generally reserved only in moderate e sever cases [12–14].

However, it appears evident how the stridor can be caused by a variety of conditions from benign, self-limited diseases to life-threatening requiring rapid intervention.

Therefore, it is necessary to identify a first-level, noninvasive tool in the management of infants with stridor in order to guide the clinician in the diagnostic-therapeutic pathway.

Recently, advances in technology have allowed the development of no invasive techniques to study respiratory function, also in uncooperative infants. In particular, tidal breathing flow-volume loop (TB-FV) and the multiple breath washout (MBW) are becoming increasingly helpful in pediatric practice in a large variety of respiratory conditions [15–18]. However, there are still few data on their application in infants with suspected airway obstruction.

In this context, we wanted to conduct a study to evaluate the role of respiratory function testing in a cohort of infants with stridor. The aims of our study were: 1) to evaluate the role of respiratory function testing (PFR) in the diagnosis and follow-up of infants with stridor; 2) to evaluate the differences between patients with inspiratory stridor and expiratory stridor; 3) to evaluate the concordance between PFR and endoscopy.

## Methods

### Study design

This was a retrospective study conducted in the Department of Pediatrics of the University “La Sapienza” of Rome from October 2021 to September 2023.

#### Participants

We enrolled infants full-term younger than 12 months with a clinical diagnosis of chronic inspiratory e/o expiratory stridor (cases) and a group of patients with negative past history for respiratory diseases (controls).

Chronic stridor is a stridor that is present at birth or shortly thereafter, not acutely. It is daily, increases during crying and not related to infection, to foreign body aspiration and iatrogenic insults.

Exclusion criteria were: age > 12 months, prematurity, patients with previous endoscopic evaluations, other comorbidities (chronic respiratory disease, cardiac diseases, genetic diseases, neuromuscular diseases).

### Procedures

Demographic characteristics and medical history including age, height, weight, breastfeeding, type of birth, family history of atopy, exposure to smoke were obtained during the first visit.

Patient cases were classified into three groups according to a clinical score [19]: mild (isolated stridor); moderate (stridor associated with cough, regurgitation or difficulty feeding); severe (stridor associated with apnea, cyanosis, growth failure).

After a first pediatric assessment, all subjects underwent a TB–FV loop study (T0); Based on morphological findings at TB-FV, we identified 4 specific patterns: pattern 1 characterized by deep fluctuations of inspiratory flow rate with normal expiratory phase (suspected extrathoracic obstruction), pattern 2 characterized by expiratory flattening with normal inspiratory phase (suspected intrathoracic obstruction), pattern 3 with involvement of both respiratory phases (suspected intra and extrathoracic obstruction) and pattern 4 with round or oval conformation ( healthy patients).

When clinically indicated, specifically patients with moderate and severe forms of stridor underwent bronchoscopy.

Patient cases underwent TB–FV loop study at follow-up, respectively 3 months (T1), 6 months (T2) and 12 months (T3) from T0.

#### Tidal breathing flow-volume loop

The TB-FV loops were recorded with a computerized infant pulmonary function device (“Exhalyzer and Spiroware”, ECO MEDICS, Bubikonerstr. 45, CH-8635 Duernten Switzerland), allowing immediate graphic visualization of flow-volume curve. The airway functional study was performed during quiet sleep, in a supine position with the head midline and the neck slightly extended to minimize airway or glottis obstruction. A face mask of size 1 or 2 was utilized according to the size and weight of each patient, in adherence with the American Thoracic Society/European.

Respiratory Society recommendations and positioned in such a way to cover the mouth and the nose, not allowing air leak [20, 21]. The machine was calibrated according to the infant’s weight and length at each test. At least three consecutive breaths were set as minimum required to have a valid test.

The main parameters of the TB-FV loop registered were tidal volume (VT), tidal volume per kg (VT/kg), inspiratory and expiratory volumes (VI and VE), inspiratory and expiratory times (Ti and Te), time-to-peak tidal expiratory flow as a percentage of total expiratory time (TPTEF/Te), peak inspiratory and expiratory flows (PIF, PEF), respiratory rate (RR) [22].

#### Bronchoscopy

Subjects were sedated with midazolam, fentanest and propofol intravenously. During the procedure hearth rate, respiratory rate, and pulse oximetry were recorded. Bronchoscopy was performed via the nasal route, using pediatric flexible bronchoscope (Pentax). Airway findings were videotaped.

### Statistical analysis

Statistical analysis was performed using SPSS statistical software (version 27, IBM, New York, USA). A descriptive analysis was performed for all the variables studied using percentage values ​​for the qualitative variables, and the mean values ​​and relative standard deviations for the quantitative variables. For the discrete variables, non-parametric univariate statistics tests were used (X2 test and Fischer’s exact test for very low frequencies). The Spearman coefficient test was used to study the correlation between quantitative variables. For concordance analysis, we used the Test Kappa of Cohen. *P* values < 0.05 were considered significant.

## Results

### Demographics and clinical characteristics

We enrolled 74 patients; 48 infants with a clinical diagnosis of chronic stridor, respectively 42 with inspiratory stridor and six with expiratory stridor (group of cases) and 26 healthy infants (group of controls). For cases, the mean age was *2.45 (*± *2.12 months); for controls 2.76 (*± *4.24) months.*

Cases were subdivided in 3 groups, according to clinical score: mild (composed by 32 patients), moderate (12 patients), severe (4 patients).

Demographics and Clinical Characteristics are summarized in Table 1.

**Table 1:** Demographics and clinical characteristics of patients enrolled

	**Cases**	**Controls**	***P* < 0.05**
**Cases/Controls, n**	48	26	
**Gender, M (%)**	32 (66.7)	16 (61.5)	0.66
**Age, Months (SD)**	2.48 ± (1.62)	2.47 ± (2.26)	0.98
**Weight, Kg (SD)**	5.12 ± (1.48)	4.90 ± (1.95)	
**Lenght, cm (SD)**	56.85 ± (8.87)	57.98 ± (5.39)	
**Breastfeeding (%)**	55.6		
**Family atopy (%)**	57.1		
**Smoke (%)**	25.4		
**Clinical score, n(%)**	Mild: 32 (66.7) Moderate: 12 (25.0) Severe: 4 (8.3)		

### Tidal breathing flow-volume loop at first evaluation (T0)

#### Comparison of cases and controls

**Fig. 1:**
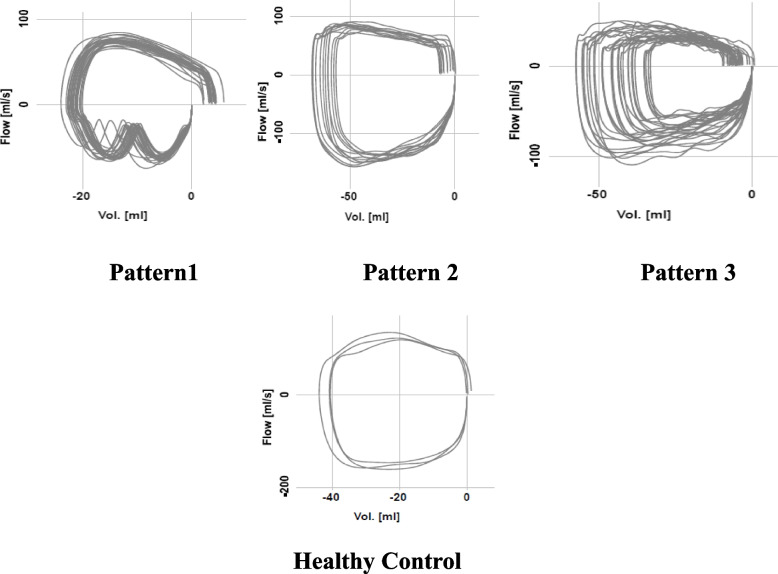
Different morphological patterns of the TV-FV loop: pattern 1: patients with inspiratory stridor; pattern 2: patients with expiratory stridor; healthy control

At first evaluation (T0), cases showed 3 different morphological patterns: pattern 1 characterized by deep fluctuations of inspiratory flow rate with normal expiratory phase (41 patients with inspiratory stridor); pattern 2 characterized by expiratory flattening with normal inspiratory phase (4 patients with expiratory stridor); pattern 3 with involvement of both respiratory phases (3 patients with bifasic stridor inspiratory and expiratory). While controls showed a round or oval conformation (Fig. 1).

Respiratory parameters obtained by recording TB–FV in the 2 group of patients are reported in Table 2. Patient cases had significantly longer inspiratory and expiratory than controls (Ti: *p* < 0.0001; Te: *p* < 0.001).

**Table 2:** Respirator parameters of TB-FV at first evaluation (T): comparison cases and controls

	**VINS**	**VESP**	**VT/Kg**	**PIF**	**PEF**	**TI**	**TE**	**TPTEF/TE**
**Cases**	43.00 ± 15.56	40.45 ± 13.94	8.20 ± 2.38	112.82 ± 50.59	100.50 ± 52.90	0.58 ± 0.17	0.66 ± 0.25	32.15 ± 12.84
**Controls**	38.40 ± 16.43	37.57 ± 16.79	7.97 ± 2.22	114.70 ± 48.25	108.77 ± 46.25	0.44 ± 0.07	0.49 ± 0.10	36.67 ± 9.54
***P* value**	0.94	0.32	0.65	0.74	0.07	**0.0001**	**0.001**	0.048

#### Comparison patients with inspiratory stridor and expiratory stridor

**Fig. 2:**
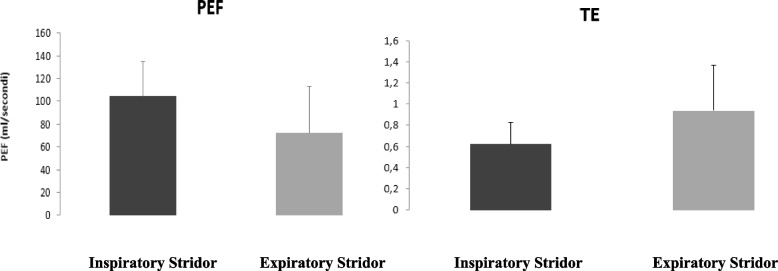
Differences of respiratory parameter between patients with inspiratory stridor and patients with expiratory stridor

Comparing respiratory parameters of patients with different type of stridor, we observed that patients with expiratory stridor had a lower peak expiratory flow and increased expiratory time than patients with inspiratory stridor (PEF: *p* < 0.023; Te: *p* < 0.004) (Fig. 2).

Furthermore, we observed that a reduced peak expiratory flow and increased expiratory time were associated with more severe clinical forms of stridor (PEF: *p* < 0.008; Te: *p* < 0.04).

#### Concordance between TB-FV loop and bronchoscopy

In our case group, 16 patients in the moderate and severe group underwent endoscopic examination.

The endoscopic diagnosis were: laryngomalacia (9 cases), subglottic hemangioma (1 case), tracheal stenosis (1 case), tracheomalacia (2 case), concomitant laryngomalacia and tracheomalacia (2 cases), suis bronchus (1 case).

**Fig. 3:**
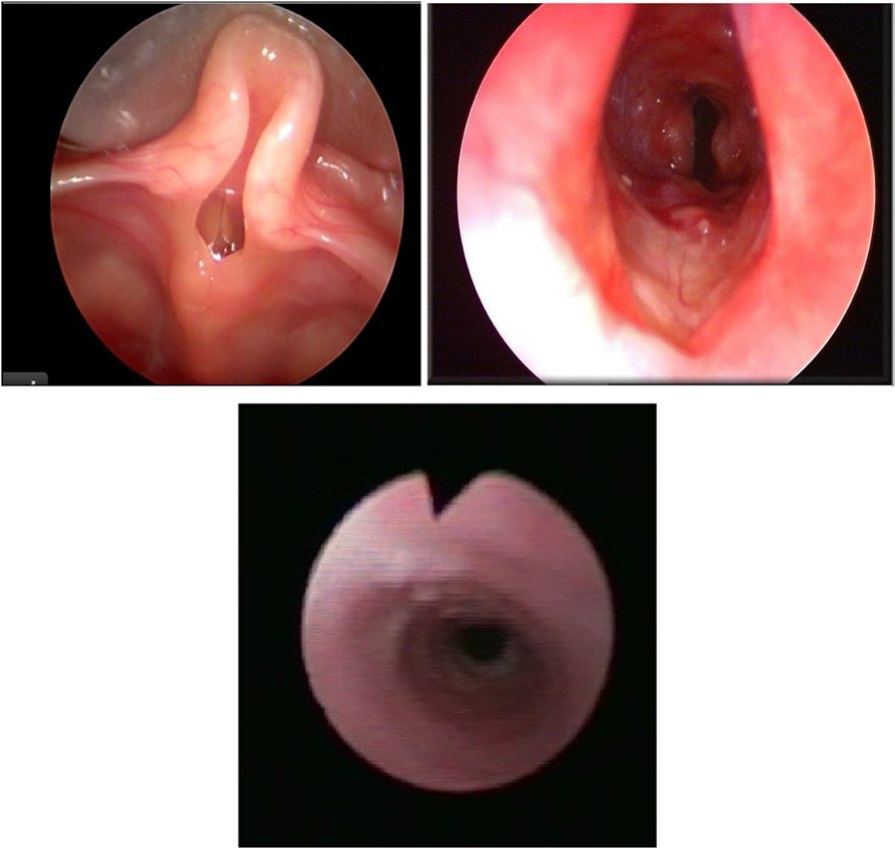
This figure includes the most the most relevant endoscopic findings: upper left shows laryngomalacia, upper right a subglottic hemangioma, below a tracheal stenosis

We demonstrated an excellent concordance between the diagnostic suspicion at the curve (intrathoracic obstruction, extrathoracic obstruction, or both) and endoscopic diagnosis (k = 0.885, *p* < 0.0001). Figure 3 shows the most relevant endoscopic findings.

### Tidal breathing flow-volume loop at follow-up

The follow-up was conducted after 3 months (T1), 6 months (T2), 12 months (T3) from T0.

We performed TB-FV in 30 patients at T1; in 24 patients at T2; in 14 patients at T3.

**Fig. 4:**
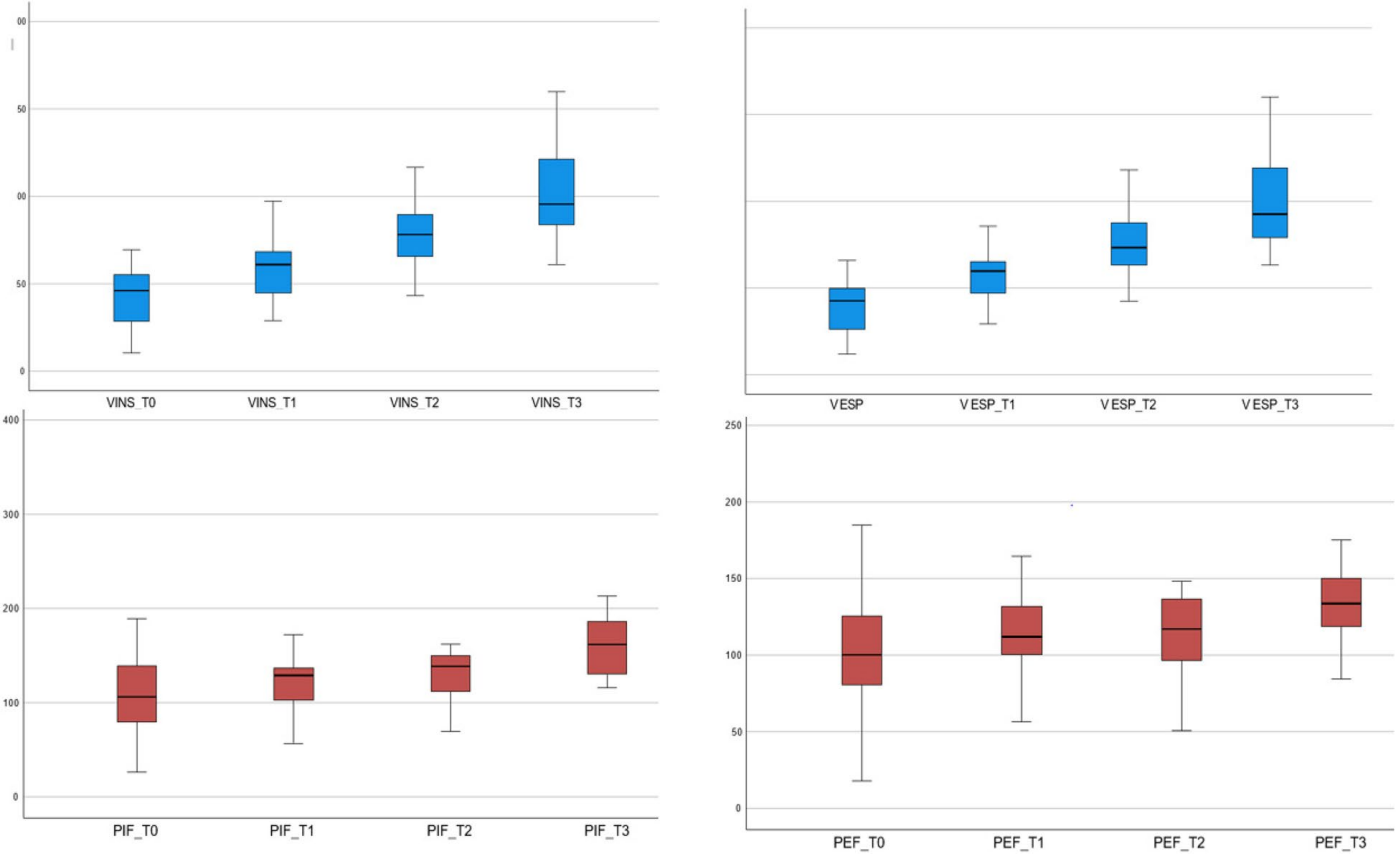
Increase of respiratory parameters (inspiratory and expiratory volume, peak inspiratory and expiratory flow) at follow-up

Table 3 report respiratory parameters of TB–FV loop at the three follow-ups. We showed a progressive increase in all respiratory parameters and a reduction of respiratory rate in accordance with staturo-ponderal growth and clinical improvement (Fig. 4).

**Table 3:** Respirator parameters of TB-FV at follow-up

	**VINS**	**V ESP**	**RR**	**VT**	**PIF**	**PEF**	**TI**	**TE**	**TPTEF/TE**
**T0**	43.0 ± 15.6	40.5 ± 13.9	50.1 ± 12.1	42.4 ± 14.4	112.8 ± 50.6	100.5 ± 32.9	0.6 ± 0.2	0.7 ± 0.3	32.2 ± 12.8
**T1**	58.6 ± 15.6	57.0 ± 14	42.5 ± 11.1	57.2 ± 15.2	123.9 ± 35.4	112.3 ± 29.3	0.7 ± 0.1	0.8 ± 0.2	27.2 ± 10.5
**T2**	77.9 ± 27.5	75.2 ± 20.5	36.2 ± 11.4	76.6 ± 23.7	147.6 ± 77.4	119.8 ± 37.3	0.8 ± 0.2	1.1 ± 0.7	29.0 ± 15.1
**T3**	102.6 ± 28.4	100.1 ± 28.3	31.3 ± 8.4	101.4 ± 28.1	159.2 ± 29.2	136.2 ± 31.5	0.9 ± 0.2	1.2 ± 0.3	27.1 ± 10.3

## Discussion

In recent years, there was growing evidence for the use of lung function testing in infancy in various respiratory diseases [23–28], but to date many clinicians still consider these methods as experimental and limited to the scope of the research.

Our findings supported the usefulness in the management of infants with suspected airway obstruction. At first evaluation, primary information derived by the graphic representation of the TB–FV. We showed different patterns depending of the site of obstruction: pattern 1 characterized by fluttering of the inspiratory phase, pattern 2 characterized by expiratory flattening, pattern 3 characterized by both components. These patterns are easily recognizable when compared with healthy infants who have a round or oval shape. In this way, only at a glance can suspicion be placed on the type of airway obstruction [29–31].

Comparing respiratory parameters recorded between case patients and controls, we showed that case patients have longer inspiratory and expiratory times than controls. These findings are consistent with the presence of airway obstruction impeding airflow, however they have low diagnostic utility because there is a large intra-subject and intersubject variability [31, 32].

Instead, interesting results derived by comparison between patients with different type of stridor; in fact, patients with expiratory stridor present a reduced expiratory peak flow and a longer expiratory time than patients with inspiratory stridor. These data in association with graphic representation of the loop add to the clinician information on the type of respiratory obstruction.

Secondary we demonstrated excellent diagnostic concordance between respiratory function tests and endoscopy. These data are in line with other studies of literature [33] and represents those could have the most important implications in clinical practice. In our study patients with pattern 1 had as endoscopic diagnosis laryngomalacia, patients with pattern 2 had as endoscopic diagnosis conditions such as: tracheomalacia, tracheal stenosis, subglottic hemangioma, patients with pattern 3 had involvement of both the larynx and the intrathoracic trachea.

In this context, Tidal breathing flow-volume loop could be a valid screening tool able to discriminate patients with isolated laryngomalacia who do not need endoscopic examination, thus reducing invasive procedures and costs; Certainly, in association with clinical evaluation and physical examination.

An innovative aspect of our study is the evaluation of respiratory parameters of the curve correlated with clinical severity. In fact, to date no significant differences in respiratory curve parameters have been described between patients with different degrees of obstruction [34]. We found that lower peak expiratory flow and longer expiratory time were associated with severe forms of stridor. This aspect is important in identifying the most at-risk patients and initiating early second-level diagnostic investigations such as endoscopy and targeted therapeutic strategies.

Finally, we performed the TB-FV at 3, 6, and 12 months of first evaluation. Few are the study present in literature that evaluated the application of the methodic at follow-up. We showed a progressive increase in all respiratory parameters and a reduction in respiratory rate in accordance with staturo-ponderal growth and clinical improvement. This approach could be helpful both in patients managed conservatively to add information to clinical examination and to reassure caregivers about the benignity and resolution of the condition, both in those undergoing surgery [35] to verify the improvement in breathing patterns and monitor future respiratory development in these infants.

Despite the many advantages of this method, it is important to underline that this new diagnostic tool presents some limitation: it can be influenced by many factors as light sleep, the timing of the last meal (because the full stomach can alter the test), the size of the mask.

Therefore, it is important to perform the test when patients are sleeping soundly, away from the last meal, and with a correctly sized mask to reduce potential bias.

This study has some limitation: the small number of patients enrolled; not all patients performed bronchoscopy and the lack of complete follow-up for all patients.

## Conclusion

Our findings confirm the validity of the use of TB-FV loop analysis in the management of infants with suspected airway obstruction. The advantages of the method (simplicity, rapidity, non-invasiveness) make it an excellent screening tool in the hands of the clinician to reduce the use of invasive procedures in mild self-limiting cases and to select the most at-risk patients early. This approach could contribute to a better use of economic resources of the health system and to improve the quality of pediatric care.

## Data Availability

The datasets used and/or analysed during the current study are available from the corresponding author on reasonable request.

## Abbreviations

PFR: Respiratory function tests
TB-FV: Tidal breathing flow-volume
